# Feature-based clustering of the left ventricular strain curve for cardiovascular risk stratification in the general population

**DOI:** 10.3389/fcvm.2023.1263301

**Published:** 2023-11-30

**Authors:** Evangelos Ntalianis, Nicholas Cauwenberghs, František Sabovčik, Everton Santana, Francois Haddad, Piet Claus, Tatiana Kuznetsova

**Affiliations:** ^1^ Research Unit Hypertension and Cardiovascular Epidemiology, KU Leuven Department of Cardiovascular Sciences, University of Leuven, Leuven, Belgium; ^2^ Division of Cardiovascular Medicine, Department of Medicine, Stanford University, Stanford, CA, United States; ^3^ KU Leuven Department of Cardiovascular Sciences, Cardiovascular Imaging and Dynamics, University of Leuven, Leuven, Belgium

**Keywords:** left ventricular strain curves, time series analysis, machine learning, risk stratification, general population

## Abstract

**Objective:**

Identifying individuals with subclinical cardiovascular (CV) disease could improve monitoring and risk stratification. While peak left ventricular (LV) systolic strain has emerged as a strong prognostic factor, few studies have analyzed the whole temporal profiles of the deformation curves during the complete cardiac cycle. Therefore, in this longitudinal study, we applied an unsupervised machine learning approach based on time-series-derived features from the LV strain curve to identify distinct strain phenogroups that might be related to the risk of adverse cardiovascular events in the general population.

**Method:**

We prospectively studied 1,185 community-dwelling individuals (mean age, 53.2 years; 51.3% women), in whom we acquired clinical and echocardiographic data including LV strain traces at baseline and collected adverse events on average 9.1 years later. A Gaussian Mixture Model (GMM) was applied to features derived from LV strain curves, including the slopes during systole, early and late diastole, peak strain, and the duration and height of diastasis. We evaluated the performance of the model using the clinical characteristics of the participants and the incidence of adverse events in the training dataset. To ascertain the validity of the trained model, we used an additional community-based cohort (*n* = 545) as external validation cohort.

**Results:**

The most appropriate number of clusters to separate the LV strain curves was four. In clusters 1 and 2, we observed differences in age and heart rate distributions, but they had similarly low prevalence of CV risk factors. Cluster 4 had the worst combination of CV risk factors, and a higher prevalence of LV hypertrophy and diastolic dysfunction than in other clusters. In cluster 3, the reported values were in between those of strain clusters 2 and 4. Adjusting for traditional covariables, we observed that clusters 3 and 4 had a significantly higher risk for CV (28% and 20%, *P* ≤ 0.038) and cardiac (57% and 43%, *P* ≤ 0.024) adverse events. Using SHAP values we observed that the features that incorporate temporal information, such as the slope during systole and early diastole, had a higher impact on the model's decision than peak LV systolic strain.

**Conclusion:**

Employing a GMM on features derived from the raw LV strain curves, we extracted clinically significant phenogroups which could provide additive prognostic information over the peak LV strain.

## 1. Introduction

Cardiovascular (CV) diseases remain the leading cause of mortality and morbidity (1). With increased life expectancy and the prevalence of risk factors, the burden of CV diseases, including heart failure (HF), continues to rise (2). The early identification of asymptomatic individuals at risk for HF and cost-effective prevention strategies are thus of paramount importance (3). In this regard, the use of advanced computational models, built upon echocardiographic information could be helpful in improving and personalizing risk stratification.

Echocardiography is a safe, non-invasive and widely used test that is considered the gold standard for assessing cardiac geometry and function (4). In addition, the emergence of speckle tracking echocardiography (STE) has facilitated the accurate assessment of myocardial deformation (strain) (5). Several studies have shown that both left atrial (LA) and left ventricular (LV) longitudinal strain are early indicators of heart dysfunction and independent predictors of adverse outcomes in the general population (6–9).

However, the majority of previous studies used only the peak LV systolic strain in the analyses, disregarding potentially important information hidden in other parts of the deformation curves (e.g., slopes and the diastolic phase). On the other hand, the integration of temporal information obtained from the entire LV strain curve may help to assess heart health more accurately. Furthermore, understanding the impact of parameters summarizing the temporal changes in a deformation curve could further pave the way for refining CV risk stratification, especially in asymptomatic individuals at risk. Nowadays powerful computational approaches could mine the complex bulk of time-series data collected in the clinic to build integrative profiles of heart health.

The importance of introducing machine learning (ML) in CV medicine has already been proven by many studies. For instance, several studies investigated the impact of supervised and unsupervised ML models in the assessment of CV health, using routinely measured echocardiographic indexes reflecting different aspects of cardiac structure and function (10, 11). At the same time, the computational ability of ML models has enabled us to explore the clinical value of time series variables obtained from echocardiography such as LV velocity and strain curves (12, 13). To our knowledge, the published studies addressing this issue were limited to patients with symptomatic HF (12, 13).

Therefore, in this study, we tested the hypothesis that by applying unsupervised learning approaches to features derived from the time-series LV strain curve, we could identify distinct strain phenogroups in the general population that associate with CV risk profiles and adverse outcomes.

## 2. Materials and methods

### 2.1. Study participants

For our analysis, we used data obtained from two general population studies, namely the Flemish Study on Environment, Genes and Health Outcomes (FLEMENGHO) (8) and the European Project of Genes in Hypertension (EPOGH) (14). The FLEMENGHO study is a longitudinal family-based population resource on the genetic epidemiology of CV phenotypes. In this study, a population sample was recruited within northeast Belgium as described elsewhere (https://flemengho.eu/en/) (8). The study was approved by the Ethics Committee of the University of Leuven (S64406) and written informed consent was obtained. In this analysis, we included 1,284 participants who have been examined in 2009–2014 and in whom LV deformation profiles were collected in .text format. We excluded 92 subjects with an atrial fibrillation or a pacemaker (*n* = 40), or with low-quality echocardiographic images for LV strain assessment (*n* = 52). Finally, recordings with a frame rate lower than 45 Hz (*n* = 7) were not taken into consideration resulting in a final dataset of 1,185 participants.

The EPOGH cohort was used to externally validate the trained model and evaluate its predictive performance. In the EPOGH study, the individuals were recruited using the same approach as in FLEMENGHO. Additionally, both studies shared the same clinical and echocardiographic protocols. Using the same exclusion criteria we finally utilized data from 545 individuals from the EPOGH cohort.

In both studies, we applied a standardized questionnaire to collect information on the participants’ medical history, lifestyle (e.g., smoking and drinking habits) and medication intake. Blood pressure (BP) was the average of five auscultatory readings obtained while the participant was seated. We defined hypertension as a systolic blood pressure higher than 140 mmHg and/or a diastolic blood pressure above 90 mmHg and/or the intake of antihypertensive drugs (15). Diabetes mellitus was defined by a self-report, a fasting serum glucose level above 126 mg/dl and/or the intake of antidiabetic medications (16).

### 2.2. Echocardiography

All participants abstained from smoking, heavy exercise and consuming alcohol or caffeinated beverages at least 3 h before the clinical examinations. The echocardiography was performed after the participant had a 15-minute rest in supine position.

#### 2.2.1. Data acquisition

As described elsewhere (17, 18) experienced physicians performed echocardiography using a Vivid 7 Pro and Vivid E9 (GE Vingmed, Horten, Norway) interfaced with a 2.5-–3.5-MHz phased-array probe. With the subject in partial left decubitus position, the observers recorded images along the parasternal long and short axes and from the apical four- and two-chamber and long axis views together with a simultaneous ECG signal. The observers recorded pulsed-wave Doppler velocities in the LV mitral and outflow tracts from the apical view. All recordings were digitally stored for off-line post-processing.

#### 2.2.2. Off-line analysis

The post-processing of echocardiographic images was performed by an experienced observer (T.K) blinded to the participants’ characteristics. The images were processed in a workstation with EchoPAC software, version 202 (GE Vingmed, Horten, Norway). We calculated LV mass using end-diastolic LV dimensions and an anatomically validated formula. LV hypertrophy was defined as LV mass index (LVMI) higher than 50 g/m^2.7^ in men and 47 g/m^2.7^ in women (15). We calculated the LV ejection fraction using LV end-systolic and end-diastolic volumes measured by the biplane method of the disks. The maximal LA volume was measured at the end of systole by the same method and was indexed to the body surface area (LAVI).

Using transmitral blood flow Doppler recordings, we measured peak early (E) and late (A) diastolic velocities, their ratio (E/A) and A flow duration. We determined the duration of the pulmonary vein (PV) reversal time during atrial systole using PV flow signal in 1,169 out of 1,185 subjects (98.6%). On tricuspid continuous Doppler recordings (if detectable), we determined the peak velocity of the tricuspid regurgitation (TR) jet at the modal frequency. From pulsed-wave Tissue Doppler Imaging (TDI) recordings, we extracted the early diastolic mitral annular velocity (e’) at the septal and lateral walls. We calculated the E/e’ ratio by dividing transmitral E peak by TDI e’ peak averaged from both acquisition sites.

Based on our previous population study (19), we classified LV diastolic dysfunction as E/e’ ≥9.5 or as borderline E/e’ between 8.5 and 9.5 combined with any of the following: low peak LA strain (< 23%), LA enlargement (LAVI ≥45 ml/m^2^), TR (> 2.5 m/s) or prolonged reverse atrial flow (i.e., mitral atrial flow ≤ reverse PV flow - 10 ms).

The LV strain curves were extracted using myocardial speckle tracking software (Q-analysis, GE Vingmed) at default settings (8, 17), which automatically tracks the motion of the myocardium. We traced the LV endocardium borders at the end-systole from the apical 4-chamber view. The full LV strain tracing of one heart cycle were saved in .txt format together with the ECG trace. In these .txt files, information regarding the start and the end of the cardiac cycle was included.

As described elsewhere (8) the intra-observer reproducibility of LV strain was calculated. The relative bias was 2.51 ± 3.02% with absolute limits of agreement ranged from 8.44% to 3.41% and reproducibility of 6.1%.

### 2.3. Outcome assessment

We compiled information on adverse outcomes in both population cohorts to assess the incidence of events with respect to the extracted LV strain clusters. Using the Belgian health registry we collected fatal events until December 2021 in the FLEMEGHO cohort. The incidence of non-fatal outcomes was assessed via a follow-up visit or a telephone interview using a standardized questionnaire. All diseases reported by the participants were cross-checked and supplemented using information obtained from general practitioners and/or regional hospitals. Adverse cardiac events comprised coronary events (myocardial infarction, acute coronary syndrome, angina pectoris/ischemic heart disease requiring coronary revascularization), HF, atrial fibrillation and pacemaker implantation. CV events included the cardiac events along with fatal and non-fatal stroke and peripheral revascularization. In our analysis, we only considered the first event per subject.

### 2.4. Cluster analysis

**Figure 1:**
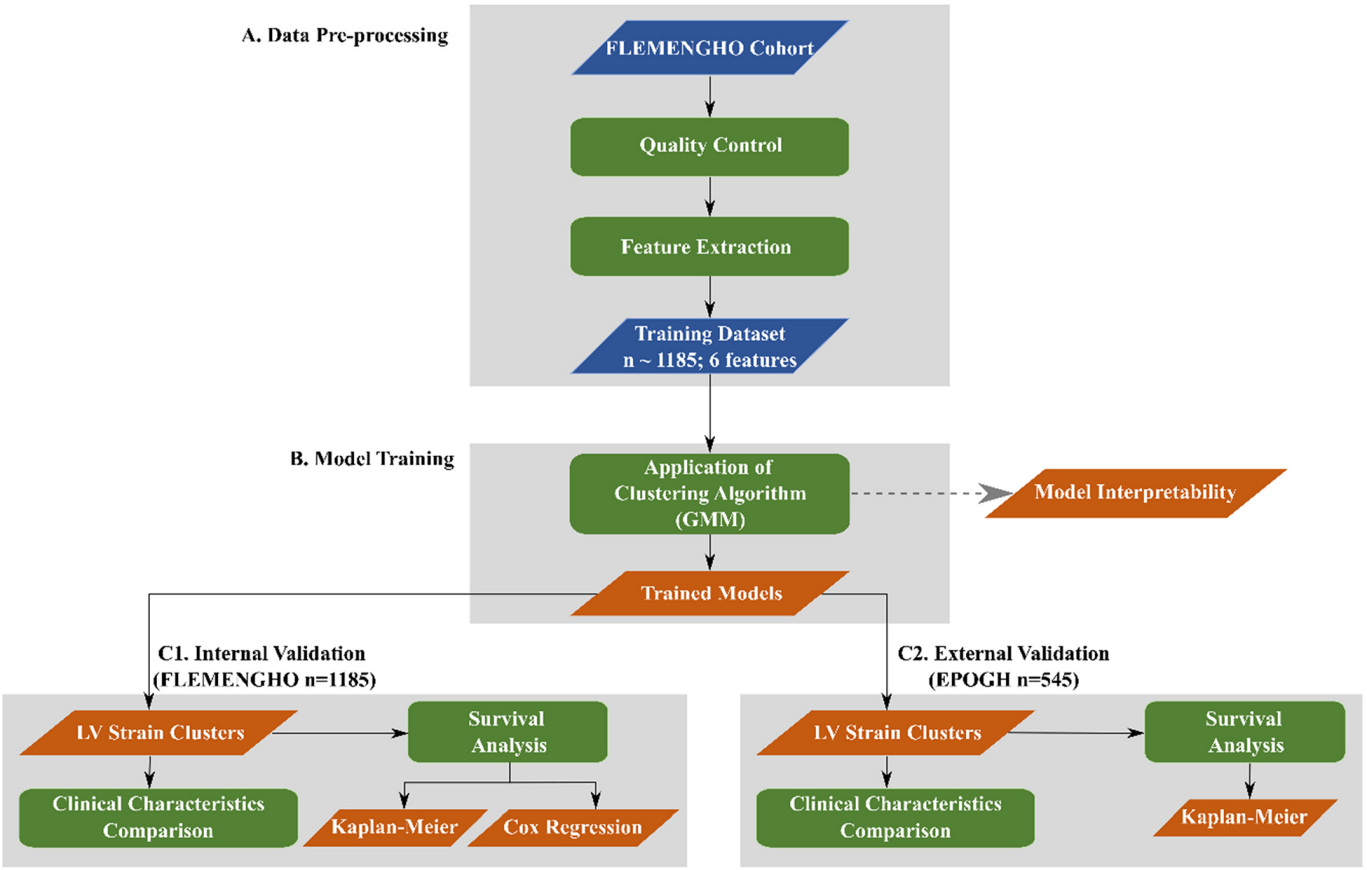
Overview of the computational pipeline. Blue and orange parallelograms illustrate the input data and the output of the processing steps, respectively. Green rectangles indicate data processing steps. The flow of the steps is represented by black arrows.

To separate the participants into phenogroups (clusters) of distinct LV deformation patterns, we performed an unsupervised learning analysis. For the implementation of the proposed approach, we used standard Python 3.9 environment (https://www.python.org) along with well-established signal processing (SciPy) (20) and scientific libraries (NumPy and Scikit-learn) (21, 22). An overview of the adopted computational pipeline is shown in Figure 1. The python scripts implementing the steps illustrated in Figure 1 are publically available at https://github.com/HCVE/LV_strain_clustering.git.

#### 2.4.1. Signal pre-processing

After a quality assurance process of the LV deformation patterns, we applied an ECG landmark-based alignment to compensate for the time offset across the different LV strain traces. The observed time shifts were due to the differences in the frame rate of the echocardiographic images and/or in the heart rate between the study participants. To perform the temporal alignment, we segmented the LV strain curves based on the cardiac cycle events, including the peaks of R- and *P*-waves and the aortic valve closure (AVC). Then we resampled the LV strain curves to match the duration of the longest recorded sequence.

Moreover, we approximated the LV strain rate by calculating the derivative of the LV strain curves. The latter was achieved by calculating the difference between the pairs of consecutive samples.

#### 2.4.2. LV feature extraction

**Figure 2:**
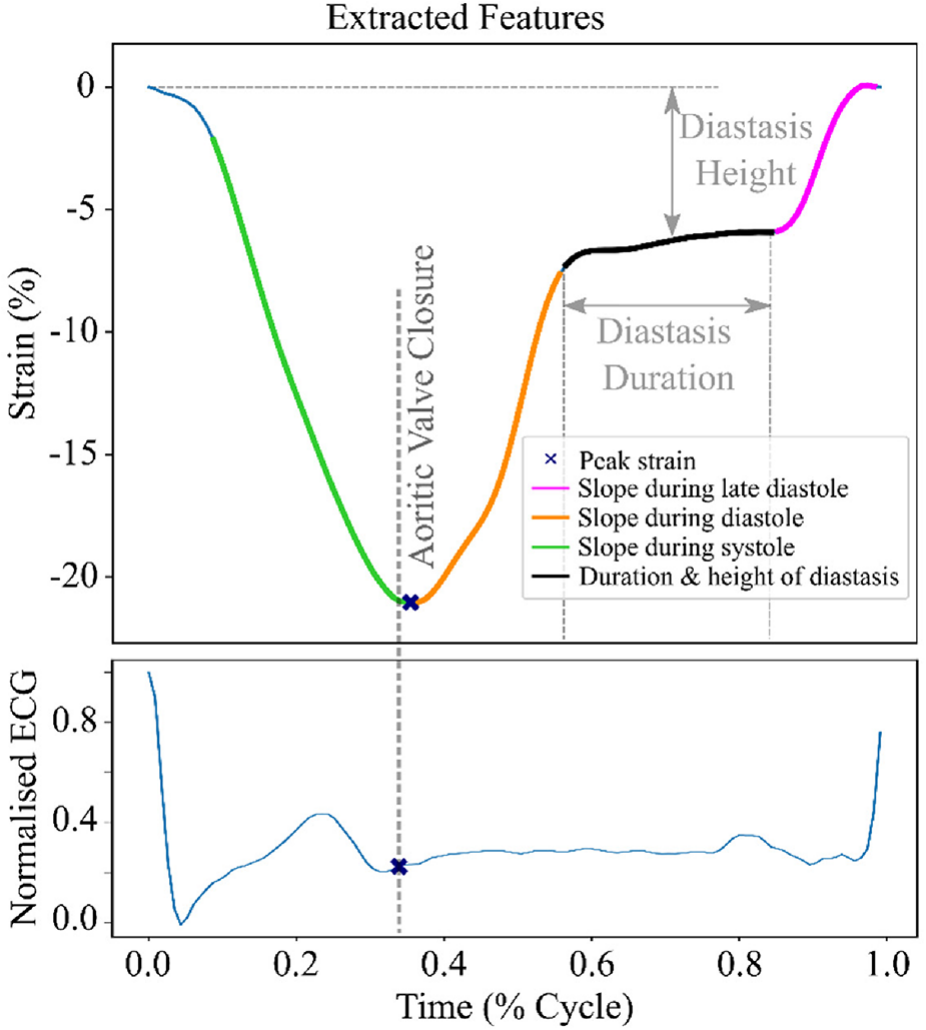
LV strain curve with the 6 extracted features used for clustering. The green region indicates the systolic phase of the heart cycle. The orange and magenta regions show the early and late diastole of the heart cycle, respectively. The black region indicates the diastasis.

To train the unsupervised model, we extracted 6 features from the raw time series LV strain curves, namely the slopes during systole, early and late diastole, the duration and the height of the diastasis and the peak LV strain (Figure 2). To identify each heart cycle phase, we first employed a piecewise linear interpolation and then, based on the obtained interpolated curve, we separated the original LV strain curve into the desired temporal regions. A detailed description of this process is given in the Supplementary data.

#### 2.4.3. Unsupervised model training

In this analysis, we opted to perform the clustering task using a Gaussian Mixture Model (GMM) algorithm trained on the 6 extracted features fitted with expectation maximization. GMM algorithm is a model-based clustering approach able to analyze complex data and identify clusters with different sizes and shapes. We used the Bayesian Information Criterion (BIC) method to determine the optimal number of clusters. After training of the clustering model on the features extracted from the FLEMENGHO cohort, we tested the model performance in the EPOGH cohort after extracting the same 6 features.

#### 2.4.4. Feature importance analysis

To better understand the clustering results and identify which features impacted the model's decision the most, we performed two feature importance analyses using the SHAP values (23) and the Random Forest algorithm. We plotted the impact of the extracted feature on the final “decision” of the GMM algorithm. A more detailed description of the model interpretability approaches is provided in the Supplementary data.

### 2.5. Statistical analysis

SAS software, version 9.4 (SAS Institute, Cary, NC, USA) was used for database management and statistical analysis. We assessed the clinical significance of the derived LV clusters (phenogroups), by comparing the clinical and echocardiographic characteristics of the participants assigned in each group in both cohorts (FLEMENGHO and EPOGH). We used Z and *χ*^2^ distributions to calculate the mean values of continuous variables and proportions of categorical variables, respectively. We also estimated the cumulative incidence of adverse events per cluster using the Kaplan-Meier method. Finally, we calculated the standardized hazard ratio using Cox regression. We adjusted the hazard ratio for baseline risk factors such as age, sex, body mass index (BMI), total cholesterol, systolic blood pressure, smoking, history of cardiac diseases and diabetes mellitus.

## 3. Results

### 3.1. Cluster analysis of LV time-series-based features

In total, 1,185 FLEMENGHO participants were included in this study, of whom 558 (47.1%) were hypertensive and from those 332 (59.5%) were on antihypertensive treatment. The mean age at baseline was 53.2 ± 15.4 years.

**Figure 3:**
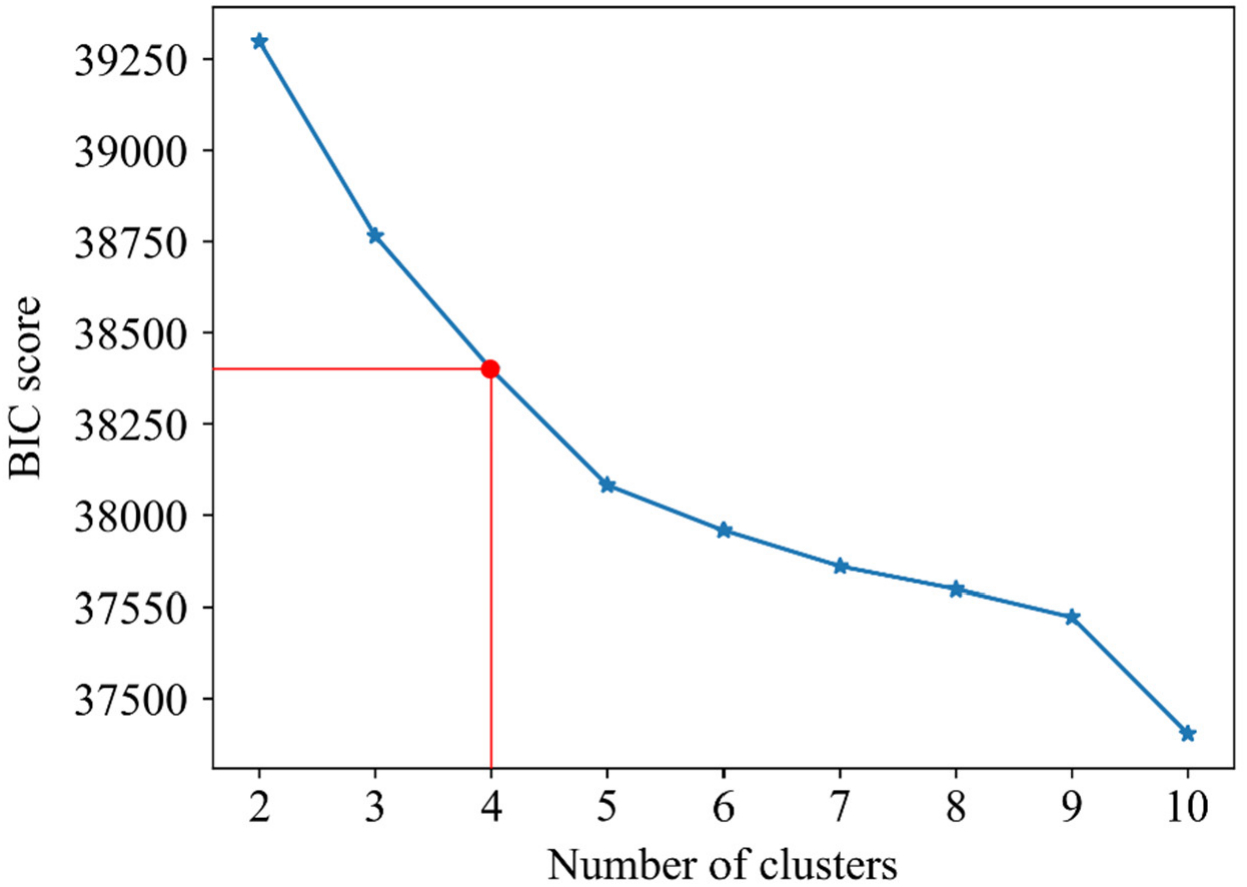
Selection of optimal number of clusters (k) for GMM based on BIC score. The point where the rate of the decrease becomes smaller, suggests the optimal number of clusters. Red lines indicate the selected number of clusters and the respective BIC score.

**Figure 4:**
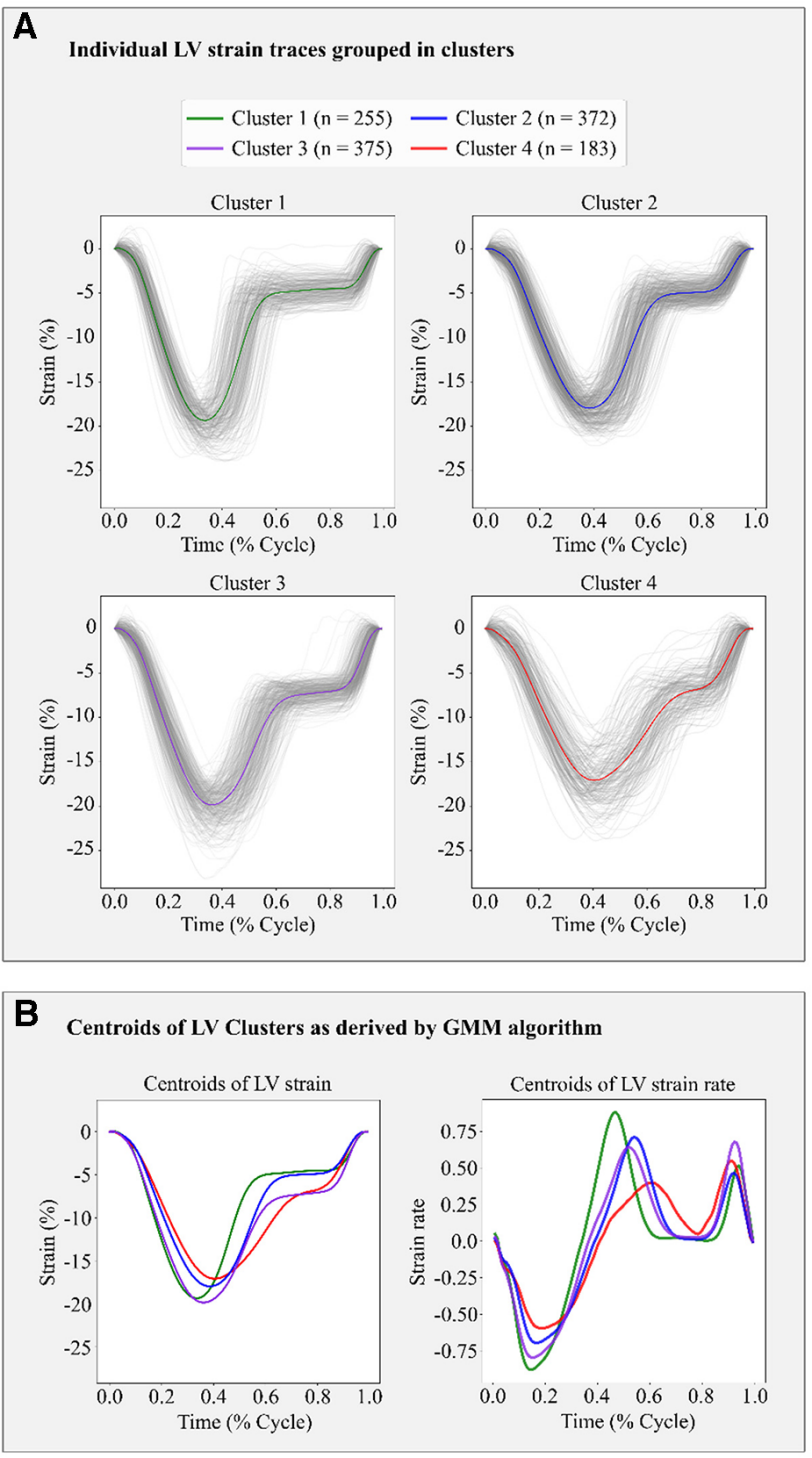
Clustering results in the FLEMENGHO cohort as derived by GMM on the 6 features extracted from the LV strain curve. (**A**) shows the individual time series LV strain curves assigned in each cluster. (**B**) presents the centroids of LV strain and LV strain rate curves of each cluster calculated as the average of the individual curves assigned to each cluster.

**Figure 5:**
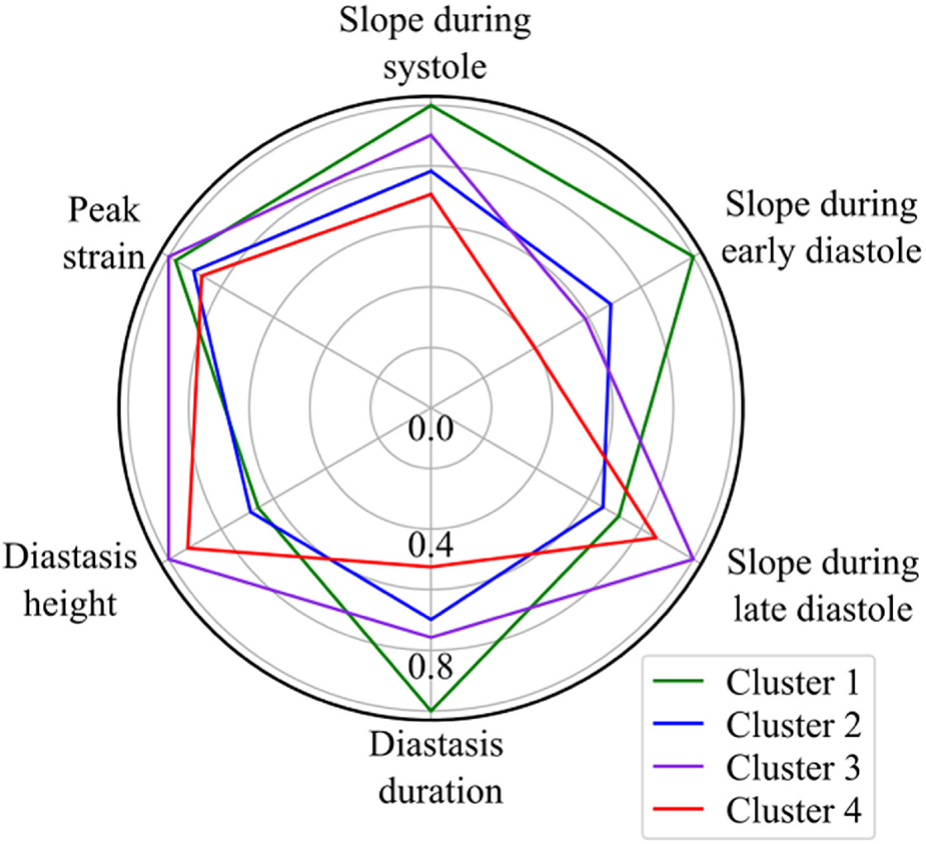
Radar chart of the 6 extracted LV strain features illustrates the superposition of the average values of parameters calculated by the trained GMM in each of the four clusters. The values are normalized with respect to the maximum value of each feature.

Based on the BIC score, the optimal number of strain clusters was between 4 and 5 (Figure 3). We opted to continue our analysis with 4 clusters, as the derived patterns showed a more meaningful partition of the LV strain curves. Figure 4 shows the individual LV strain curves per cluster along with their respective cluster centroids. We observed substantial differences between strain clusters during the diastolic phase of the heart cycle along with some differences in the peak LV strain (Figure 5).

**Figure 6:**
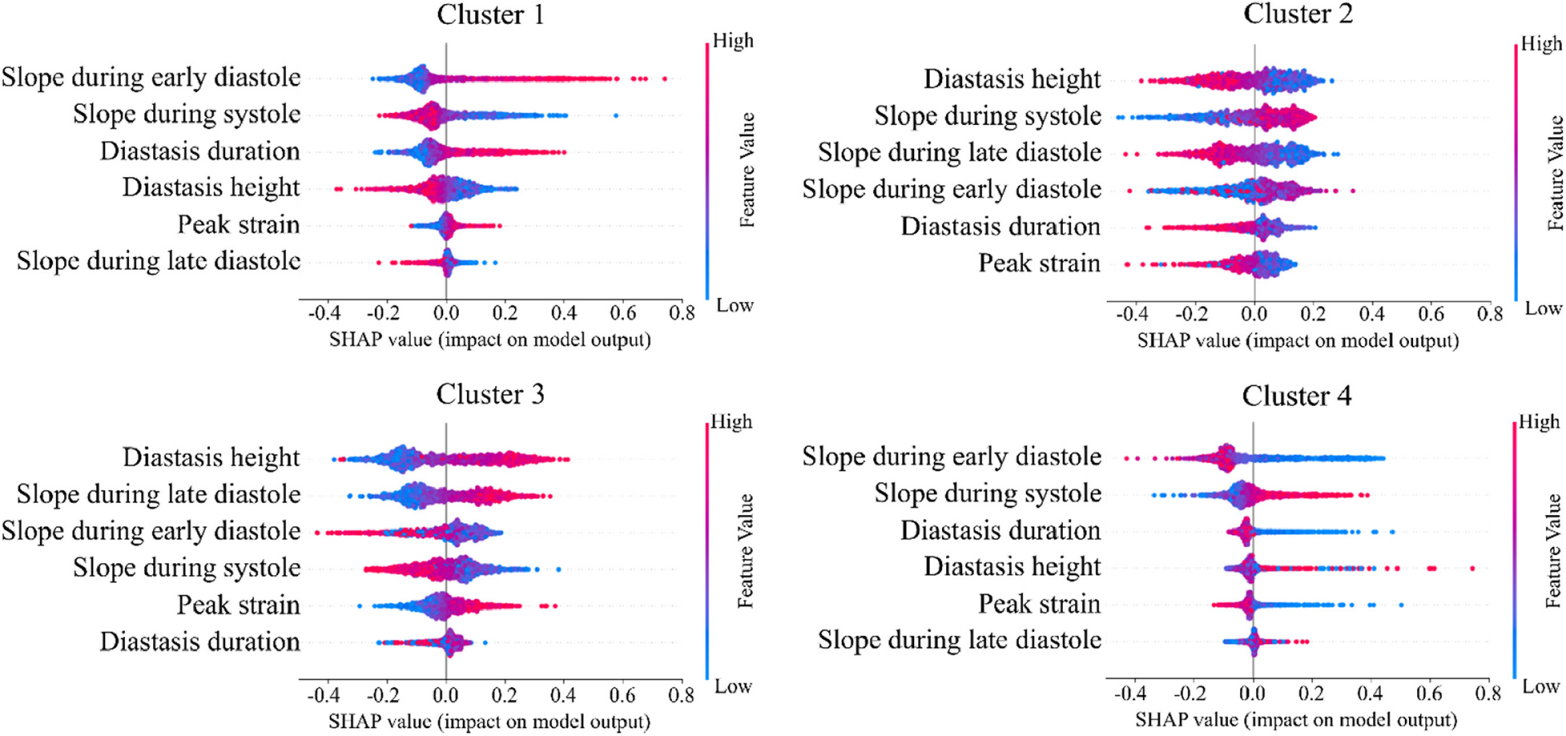
SHAP analysis per LV strain cluster. The features are ordered from the most to the least important for clustering analysis. High impact indicates that LV strain curves with the indicative feature values have higher probability to be assigned to the respective cluster.

The importance of diastole for the clustering of LV strain curves was also supported by the feature importance analysis using SHAP values (Figure 6). Indeed, for cluster 1, the most important feature corresponded to the slope during early diastole, showing that the LV strain curves with higher slopes (i.e., more rapid change) had a high probability belonging to this cluster. For clusters 2 and 3, diastasis height was the most important feature for clustering, followed by the slopes. On the other hand, cluster 4 was characterized by the smallest slopes during early diastole and systole (i.e., less rapid change), and the shortest duration of diastasis. Finally, for all clusters the peak LV strain was one of the least important features with a clear impact only for cluster 1 (high peak LV strain) and cluster 4 (low peak LV strain).

**Figure 7:**
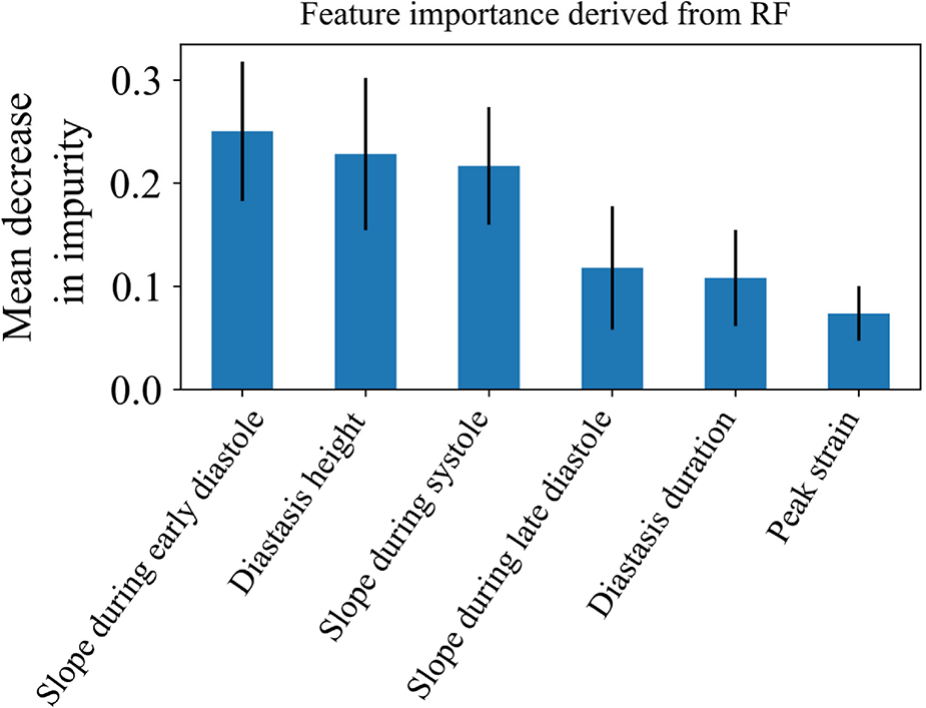
Feature importance of the 6 LV strain features used for clustering as calculated by the random forest model. The model was trained using the clustering assignments as the ground truth labels.

In addition, we trained a Random Forest model using the clustering assignments as labels, which allowed us to retrieve the feature importance, calculated as the decrease in impurity (Figure 7). The results confirmed that the most important features were those incorporating the temporal information hidden in the LV strain curves, such as the slopes during systole and early diastole along with the height of the diastasis. Thus, both SHAP and Random Forest approaches indicated that the most important features for strain clustering belonged to the diastolic phase of the cardiac cycle and the slope during systole.

### 3.2. LV strain clusters and CV risk factors

Table 1 shows the clinical and echocardiographic characteristics of the individuals belonging to each cluster. The comparison across all clusters revealed significant differences in age and heart rate (Table 1). Cluster 1 showed the most favorable CV profile, with the lowest blood pressure and the lowest percentages of hypertensive subjects and subjects with history of cardiac disease. Also, cluster 1 had the lowest prevalence of LV diastolic dysfunction with respect to the rest of the phenogroups. On the other hand, participants assigned to cluster 4 showed the worst CV profile with elevated blood pressure and high prevalence of LV hypertrophy and LV diastolic dysfunction (Table 1). In LV strain cluster 3, the reported values were in between those of clusters 2 and 4.

**Table 1:** Clinical characteristics of FLEMENGHO participants by LV strain clusters.

Characteristic	Cluster 1 (*n* = 255)	Cluster 2 (*n* = 372)	Cluster 3 (*n* = 375)	Cluster 4 (*n* = 183)
Anthropometrics				
Females, *n* (%)	136 (53.33)	187 (50.27)	197 (52.53)	88 (48.09)
Age, year	42.71 ± 12.84	46.84 ± 14.1^a^	61.58 ± 10.89^a^^,^^b^	63.85 ± 12.88^a^^,^^b^^,^^c^
Body mass index, kg/m^2^	24.9 ± 3.84	26.29 ± 4.11^a^	27.35 ± 4.16^a^^,^^b^	28.1 ± 4.26^a^^,^^b^^,^^c^
Systolic pressure, mm Hg	121.85 ± 13.36	128.2 ± 14.89^a^	136.7 ± 16.14^a^^,^^b^	141.76 ± 18.26^a^^,^^b^^,^^c^
Diastolic pressure, mm Hg	77.57 ± 8.94	82.15 ± 9.34^a^	83.1 ± 9.38^a^	86.16 ± 10.7^a^^,^^b^^,^^c^
Heart rate, beats/min	57.58 ± 7.82	65.41 ± 8.6^a^	61.79 ± 8.19^a^^,^^b^	69.69 ± 10.01^a^^,^^b^^,^^c^
Questionnaire data				
Current smoking, *n* (%)	50 (19.61)	64 (17.2)	38 (10.13)^a^^,^^b^	24 (13.11)
Drinking alcohol, *n* (%)	119 (46.67)	148 (39.78)	142 (37.87)^a^	59 (32.24)^a^
Hypertensive, *n* (%)	55 (21.57)	131 (35.22)^a^	235 (62.67)^a^^,^^b^	137 (74.86)^a^^,^^b^^,^^c^
Treated for hypertension, *n* (%)	28 (10.98)	57 (15.32)	158 (42.13)^a^^,^^b^	79 (43.17)^a^^,^^b^
History of cardiac disease, *n* (%)	3 (1.18)	15 (4.03)^a^	40 (10.67)^a^^,^^b^	29 (15.85)^a^^,^^b^
History of diabetes mellitus, *n* (%)	3 (1.18)	13 (3.49)	28 (7.47)^a^^,^^b^	20 (10.93)^a^^,^^b^
Biochemical data				
Serum creatinine, mmol/L	70.73 ± 12.26	73.03 ± 18.75	76.39 ± 23.54^a^^,^^b^	78.92 ± 21.51^a^^,^^b^
Total cholesterol, mmol/L	4.88 ± 0.93	4.93 ± 0.87	4.92 ± 0.94^a^	5.17 ± 0.98^a^^,^^b^^,^^c^
Echocardiography				
LV structure				
LV internal diameter, (cm)	5.06 ± 0.44	4.98 ± 0.4^a^	5.01 ± 0.44	4.96 ± 0.5^a^
Relative wall thickness	0.35 ± 0.04	0.37 ± 0.05^a^	0.39 ± 0.05^a^^,^^b^	0.42 ± 0.06^a^^,^^b^^,^^c^
LV mass index, (g/m)	86.84 ± 17.91	85.9 ± 17.11	96.59 ± 21.09^a^^,^^b^	100.8 ± 24.74^a^^,^^b^^,^^c^
LV hypertrophy, *n* (%)	31 (12.16)	39 (10.48)	110 (29.33)^a^^,^^b^	69 (37.7)^a^^,^^b^^,^^c^
LV systolic function				
LV end-systolic volume index, ml/m^2^	21.38 ± 5.2	20.99 ± 5.04	19.71 ± 5.26^a^^,^^b^	20.77 ± 5.51^c^
LV end-diastolic volume index, ml/m^2^	53.91 ± 10.75	51.43 ± 10.06^a^	51.49 ± 10.25^a^	49.37 ± 11.27^a^^,^^b^^,^^c^
Stroke volume index, ml/m^2^	32.54 ± 6.92	30.44 ± 6.35^a^	31.78 ± 6.63^b^	28.6 ± 7.19^a^^,^^b^^,^^c^
Ejection fraction, %	60.34 ± 5.06	59.29 ± 5.18^a^	61.88 ± 5.79^a^^,^^b^	58.11 ± 6.42^a^^,^^b^^,^^c^
Peak LV longitudinal strain, %	19.83 ± 1.8	18.39 ± 1.5^a^	20.38 ± 1.97^a^^,^^b^	17.74 ± 2.48^a^^,^^b^^,^^c^
LV diastolic function				
E/A ratio	1.79 ± 0.5	1.38 ± 0.42^a^	1.01 ± 0.27^a^^,^^b^	0.84 ± 0.3^a^^,^^b^^,^^c^
e' peak, cm/s	13.25 ± 2.95	11.43 ± 3.05^a^	8.58 ± 2.3^a^^,^^b^	7.0 ± 2.55^a^^,^^b^^,^^c^
E/e’ ratio	6.23 ± 1.39	6.52 ± 1.72^a^	7.92 ± 2.41^a^^,^^b^	8.89 ± 3.81^a^^,^^b^^,^^c^
LV diastolic dysfunction, *n* (%)	5 (1.96)	23 (6.18)^a^	65 (17.33)^a^^,^^b^	67 (36.61)^a^^,^^b^^,^^c^

Values are mean (±SD) or number of subjects (%). LV hypertrophy was a LV mass index of 52 g/m^2.7^ in men and 45 g/m^2.7^ in women or more. Significance for between-phenogroups differences.

^a^ *P *< 0.05 vs. Cluster 1.

^b^ *P *< 0.05 vs. Cluster 2.

^c^ *P *< 0.05 vs. Cluster 3. A indicates late peak diastolic velocity of transmitral blood flow; E, early peak diastolic velocity of transmitral blood flow; e’, early peak diastolic myocardial velocity; LV, left ventricular.

### 3.3. LV strain clusters and adverse events

In the FLEMENGHO cohort, the median follow-up time was 9.1 years (5th–95th percentile, 2.9–11.8). A total of 116 participants experienced at least one adverse CV event over 10,291 person-years of follow-up (11.3 events/1,000 py). For cardiac events, 81 participants experienced at least one adverse event resulting in a 7.7 events per 1,000 person-years.

**Figure 8:**
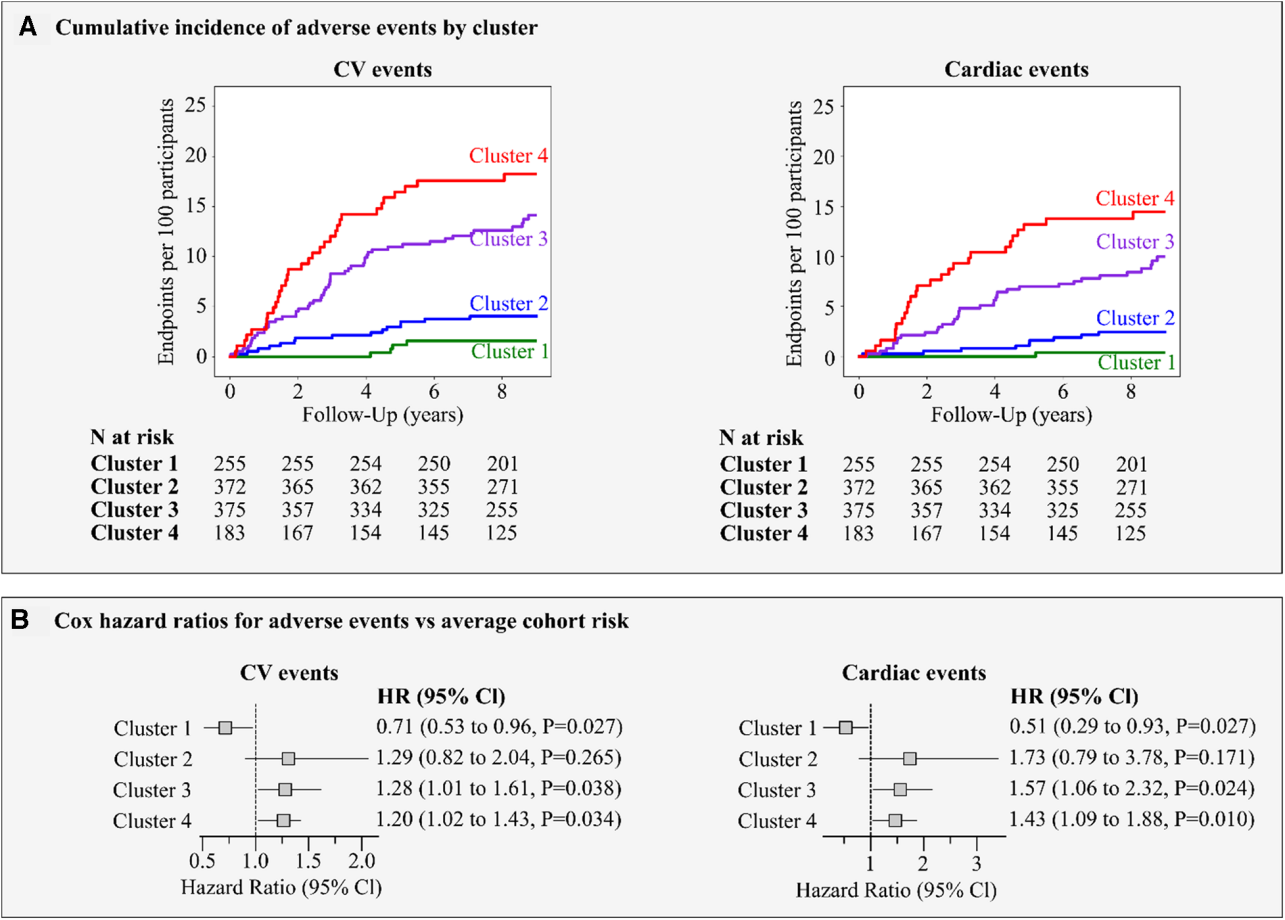
Risk for major adverse events by LV strain cluster. (**A**) shows the incidence of adverse CV and cardiac events per cluster. (**B**) presents the Cox regression hazard ratios (95% Cl) for CV and cardiac events. The hazard ratios express the risks in clusters compared to the average risk in the whole cohort and adjusted for age, sex, body mass index, smoking, blood pressure, total cholesterol, history of diabetes and cardiac diseases.

The cumulative incidence of CV and cardiac outcomes by LV strain cluster is illustrated in Figure 8, left panel A. In cluster 1, we observed a low risk for CV events with only 4 events (1.7/1,000 person-years) while strain clusters 3 and 4 showed a high risk with 55 events (17.3/1,000 person-years) and 37 events (24.9/1,000 person-years), respectively. Intermediate CV risk was observed for cluster 2 (20 events; 6.1/1,000 person-years). The same pattern was observed for cardiac events (Figure 8, right panel A).

Figure 8, panel B illustrates the adjusted hazard ratios expressing the risk in each cluster compared with the average risk in the whole cohort. In strain clusters 3 and 4, the adjusted risk was significantly higher than the average risk for CV (28% and 20%, *P* ≤ 0.038) and cardiac (57% and 43%, *P* ≤ 0.024) events respectively, whereas in cluster 1, the risk was significantly lower by at least 30% (*P* = 0.027) for all events (Figure 8, panel B). Moreover, including the LVMI and the E/e’ ratio in the Cox model, strain clusters 3 and 4 remained significant in predicting both CV (27% and 21%, *P* ≤ 0.044) and cardiac events (55% and 44%, *P* ≤ 0.028).

Although we observed the higher cumulative incidence of adverse events in subjects with an abnormal peak LV systolic strain belonging to quartile 4 (<18%) as compared to those with normal peak LV strain, the adjusted risk for adverse events was not significant (*P* = 0.29; Supplementary Figure S1).

### 3.4. External validation cohort

**Figure 9:**
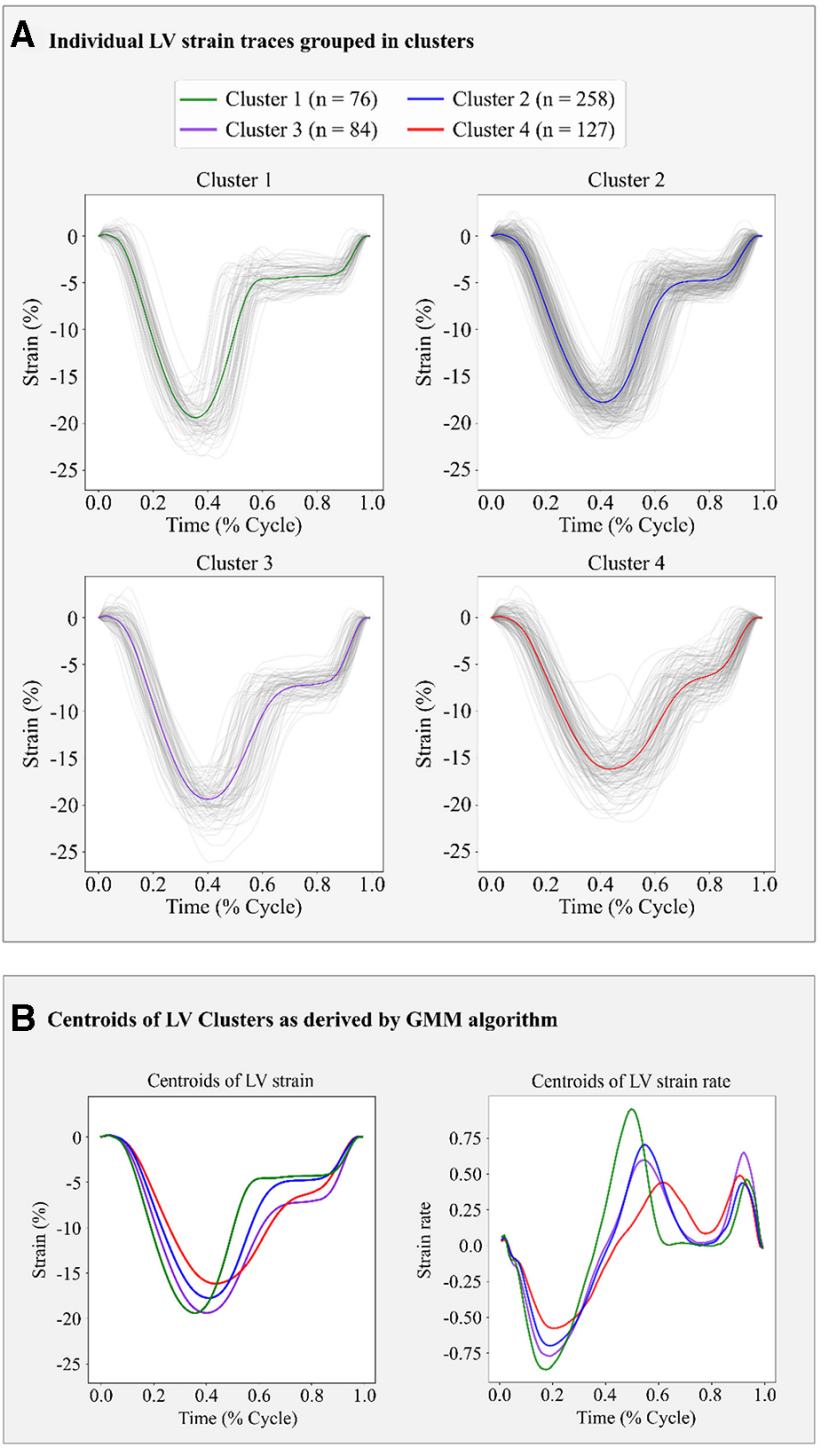
Clustering results of LV strain using the GMM trained on FLEMENGHO data on the 6 extracted features in the EPOGH cohort. (**A**) illustrates the individual strain curves assigned in each cluster. (**B**) depicts the centroids of LV strain curve and LV strain rate curve of each cluster calculated as the average of the curves assigned in each cluster.

A total of 545 EPOGH participants were included in the external validation cohort, with 309 (56.7%) being females. EPOGH cohort comprised younger participants (38.8 ± 14.4 years), with lower prevalence of hypertension (29.9%), fewer person-years of follow-up (6,070) and reported CV adverse events (*n* = 45) than the FLEMENGHO cohort. For evaluation of the trained model and to ascertain its clinical significance, we applied the GMM using the same 6 features extracted from the LV strain traces of the EPOGH cohort (Figure 9). Overall, in this validation cohort, we observed differences in clinical and echocardiographic characteristics between the LV strain clusters that were similar to those in FLEMENGHO participants (Supplementary Table S1). Also, the cumulative incidence of CV events was the highest in cluster 4 (20 events; 13.1/1,000 person-years) followed by cluster 3 (10 events; 10.5/1,000 person-years) as compared to clusters 1 (2 events; 2.5/1,000 person-years) and 2 (13 events; 4.7/1,000 person-years).

## 4. Discussion

In this analysis, we utilized an unsupervised ML model on data from community-dwelling participants to separate LV deformation patterns into phenogroups (clusters) with significant clinical relevance. Applying GMM on features derived from time series LV strain curves, we identified four groups of distinct patterns related to different CV risk profiles. Across these four strain clusters we demonstrated significant differences in age, blood pressure and heart rate distribution. Cluster 1 comprised the youngest participants with a low prevalence of CV risk factors, whereas subjects assigned to cluster 4 showed the most unfavorable CV risk profile with a higher prevalence of hypertension, LV hypertrophy and diastolic dysfunction. In strain cluster 3, the prevalence of CV risk factors was between that of clusters 2 and 4. Survival analysis and adjusted hazard ratios showed that participants in clusters 3 and 4 had the highest risk of developing adverse events as compared to the average population risk.

The prognostic value of peak LV longitudinal strain (or global longitudinal strain, GLS) has already been reported in several studies (24, 25). For instance, Sengelov et al. showed that peak LV systolic strain assessed by echocardiography was an independent predictor for all-cause mortality in patients with HF with reduced ejection fraction (24). Furthermore, a few longitudinal studies in the general population reported that lower GLS was associated with a higher risk of developing CV and cardiac events independent of traditional CV risk factors (8, 26, 27).

The recent developments in ML algorithms have created new possibilities in processing complex clinical and echocardiographic data for better prognostications and risk stratification in patients and in the community. For example, Shah et al. (28) applied GMM to identify distinct phenogroups in symptomatic patients with HF with preserved ejection fraction. Using 46 features, such as demographics, clinical characteristics, biochemical and echocardiographic indexes, the authors categorized participants into 3 phenogroups which associated with the risk of HF hospitalization. In another study, to improve CV risk stratification in the general population, Sabovčik et al. (11) also employed GMM to identify distinct echocardiographic phenogroups. The authors showed that 3 distinguished phenogroups had significant differences in the risk of developing adverse CV events. Of note, the majority of these studies assessed the prognostic value of discrete echocardiographic indexes including the peak LV strain, disregarding potentially valuable temporal information hidden in the shape of LV strain curves during the cardiac cycle.

On the other hand, studies that investigated the prognostic value of LV strain curves in the temporal domain, are limited in distinguishing symptomatic patients with heart diseases from healthy individuals. For instance, Tabassian et al. investigated the value of the temporal information hidden in segmental (29) and global (30) LV longitudinal strain to detect abnormal changes in LV mechanics and identify patients with symptomatic HF. Similarly, in a small cross-sectional study, Loncaric et al. (31) identified ML-based patterns associated with cardiac remodeling due to pressure overload in 189 patients with hypertension. Using a two-steps unsupervised ML approach including hierarchical clustering, the authors distinguished hypertensive patients from the healthy participants based on tissue and blood-pool velocity and deformation profiles during the whole cardiac cycle (31).

Previously, we demonstrated the significance of time series analysis of deformation profiles of the LA in the general population (32). Using two different clustering approaches we showed that the incorporation of the whole LA deformation patterns provides incremental predictive information over the current practice that considers only peak LA reservoir strain. Consequently, the present study extends the application of unsupervised ML modelling in the general population using the whole spectrum of LV deformation curves. By clustering features that incorporate temporal information such as the slopes of LV curve during systole or early and late diastole we were able to separate participants into distinct phenogroups associated with different clinical characteristics and risk profiles. Hence, the developed model could provide the normal patterns of LV strain curves derived from the general population as well as distinguish participants at CV high risk. This, in turn, could facilitate early intervention and improve risk management hindering the progression of cardiac dysfunction.

Along these lines, the derived centroids of each cluster could serve as templates to identify the normal or abnormal LV deformation patterns. For example, low systolic and diastolic slopes, as observed in the cluster 4 pattern, mean slow emptying and filling of the LV, respectively, which could be indicative of increased myocardial workload and higher LV stiffness. Furthermore, a shorter duration of diastasis would indicate that active LV filling during LA contraction occurs earlier, pointing out a shorter period of LV relaxation.

In addition, in this study, we demonstrated that we could retrieve the LV strain rate by calculating the derivative of LV strain. Of note, LV strain rate showed well-separated patterns for each cluster as illustrated by their respective centroids. Although this method requires further research, its application in a clinical setting could lead to a simpler and transparent manner of extracting LV strain rate which could supplement LV strain temporal data.

Another important aspect of our ML analysis is the interpretability of the developed models. By understanding the “decisions” of these non-linear models, their “black-box” characteristic is reduced promoting a better understanding of the pathophysiology of cardiac dysfunction. Consequently, interpretability accelerates and eases the adoption of ML models in the medical field. To improve the interpretability of our clustering results, we applied SHAP values and Random Forest to determine the impact of the derived features on the final construction of each strain cluster. Our analysis showed that the slopes of the LV strain curves during systole or early diastole and/or features that summarize diastasis contributed the most to the formation of the clusters. For instance, cluster 4 was characterized by LV patterns with the lowest slope during early diastole and systole, the shortest diastasis, and the smallest LV peak strain. Consequently, the incorporation of diastolic phase of the LV strain curve analysis could improve the further delineation of CV risk in patients.

While we acknowledge that using discrete cut-off values to categorize a patient's CV risk is self-explanatory and aligns with current practice, we emphasize that the objective of this study is to construct a ML model that could be used as a decision support tool by clinicians, additionally to the current practice. This allows to categorize a patient to a particular risk group based on the whole information derived from LV strain curve (in this case low-, low/intermediate-, intermediate/high-, high- risk). Therefore, this study might pave the way to integrate ML models into commercial software solutions used for strain analysis. However, a more extensive validation of the model using recordings from diverse population and patients cohorts should be performed before the clinical translation of the study findings.

### 4.1. Limitations

We recorded the LV deformation curves using speckle tracking on echocardiographic images. Of note, during the post-processing of the images, the region of interest in which speckle tracking was performed could be adjusted by an observer. Hence, the recordings used throughout our analysis were susceptible to measurement errors. Next, to define LV diastolic dysfunction in our study, we used outcome-derived population-based criteria instead of ASE criteria. As we previously reported, the prevalence of LV diastolic disfunction according to the ASE criteria was lower (1.85%) and therefore they might be less sensitive in general population settings (19). However, future studies are needed to evaluate whether epidemiologically based thresholds for diastolic parameters will better identify asymptomatic subjects at risk. Also, we included a few cases of pacemaker implantation (*n* = 9) as adverse events in our outcome analysis. Of note, most of the patients with pacemaker implantation additionally experienced other CV adverse events, such as heart failure, stroke, atrial fibrillation, etc. Therefore, although the correlation between conduction abnormalities and strain pattern is not clear, it is highly unlikely that including these events can confound the outcome results because these patients still remain in the analysis due to other adverse events. Additionally, the EPOGH cohort which we used for validation of our model included fewer participants than FLEMENGHO. On the other hand, in both datasets our analysis showed that the derived LV clusters contain clinically relevant information. We could reinforce our findings by further evaluation of the trained model on other patient or community-based datasets with available time series of LV strain curves. Finally, the BIC score did not provide conclusive results regarding the optimal number of clusters. The final selection of this parameter was performed by a visual inspection of the clustering results in the FLEMENGHO cohort for the two best values according to BIC.

### 4.2. Conclusions

Overall, we showed that unsupervised learning methods on features derived from time series LV strain curves identified clinically meaningful phenogroups which could provide additive prognostic information over the peak LV strain. All clusters revealed considerable differences in the slopes and the diastolic phase of the cardiac cycle suggesting that the introduction of diastole in the evaluation of LV strain curves could add valuable prognostic information. This could lead to the fine-tuning of CV risk stratification and consequently improve the identification of early stages of cardiac dysfunction. In addition, we provided the normal patterns of LV strain curves derived from the general population.

## Data Availability

The data analyzed in this study is subject to the following licenses/restrictions: The use of database should be approved by the PI of the study. Requests to access these datasets should be directed to Prof Tatiana Kuznetsova, tatiana.kouznetsova@kuleuven.be.
