# Supplementary material for: Feature-based clustering of the left ventricular strain curve for cardiovascular risk stratification in the general population
Source: Front Cardiovasc Med. 2023 Nov 30;10:1263301. doi: 10.3389/fcvm.2023.1263301 (PMC10720328; doi:10.3389/fcvm.2023.1263301)

Supplementary Material

Feature-based clustering of the left ventricular strain curve for cardiovascular risk stratification in the general population

# Supplementary Data

## Feature Extraction

Before the application of the selected unsupervised ML algorithm a feature extraction process was executed. This procedure consisted of three steps:

The first step incorporated a piecewise linear interpolation to approximate the LV strain curve with linear segments. Our aim was to find the best approximation with the lowest complexity possible (i.e. the fewest number of segments). To achieve that, we iteratively increased the number of segments into which we separated the initial strain curve. The lowest number of segments was selected to be seven (range: 7-15). The minimum and maximum value of the number of segments were selected empirically. This iterative procedure terminated if the number of segments reached the maximum number provided by the user or if the increase in the number of segments didn’t result in a decrease in the Akaike Information Criterion.

Using the approximation of the fitted model, we identified the regions of interest corresponding to different phases of the heart cycle, namely systole, diastole, diastasis and late diastole. Particularly, systole was approximated as the region with large positive differences. Diastole was the region with large negative differences occurring before the 85% of the heart cycle. Diastasis was defined as the region with approximately zero change in the strain and finally the late diastole was the region with large negative differences at the end of the heart cycle.

After the isolation of each region, we calculated the slopes during systole, diastole and late diastole by fitting a linear regression model in each phase separately. The height of the diastasis was approximated as the average value of the strain during this phase and the duration was calculated as the difference between the diastasis’s starting and ending time point.

To fit the piecewise linear interpolation model we used the absolute values of the strain curve.

## SHAP Values

Shapley additive explanations (SHAP) is a method based on cooperative game theory and it is used to explain individual predictions of models. The interpretability of a model’s prediction is performed by calculating the contribution of each input feature to the final decision of the model.

To explain the trained model and understand how each of the six extracted features influenced the selected clustering model the most, we used a model-agnostic approximation called Kernel SHAP. This method iteratively changes the values of the features included in the model and calculates the change in the model output. The Kernel SHAP algorithm can be summarized into five steps:

1. Sample $z_{k}^{'}\in\left\{ 0, 1 \right\}^{M}, k\in\{1, \ldots, K\}$ (1 = feature is present, 0 = feature is not present)
2. Get prediction for each $z_{k}^{'}$by first converting to the original feature space and then applying model $\hat{f}= \hat{f}(h_{x}(z_{k}^{'}))$
3. Compute the weight for each $z_{k}^{'}$ with the SHAP kernel
4. Fit weighted linear model
5. Return Shapley values (i.e. the coefficient from the linear model)

To investigate the importance of the features included in the clustering algorithm, we selected to model the probability of belonging to a specific cluster. Therefore, in the respective figure of SHAP values, LV strain curves with high positive impact feature values have higher probabilities to belong to the cluster under investigation.

Despite the advantages of “deciphering” the clustering model, this method has one important limitation. The procedure of feature permutation includes the random sampling from the feature values. Therefore, values from the marginal distribution could be sampled. This means that in case of correlated features the agnostic model may put too much weight on unlikely feature values and hence produce unreliable results.

## Random Forest Feature Importance

Random Forest (RF) algorithm is a supervised machine learning model that comprises several “weak” Decision Trees classifiers. Therefore, to train such a model we first need to define the labels of the training examples, which in our study were the labels of the cluster that each participant was assigned to. To train the RF algorithm we split the available data into a training and test set with a ratio of 80:20. Then using the training set we applied a nested cross validation to define the optimal values of the model’s parameters and finally we measured the prediction accuracy in the test set to ascertain that the trained model could adequately approximate the initial clustering model. Eventually, the prediction performance of the RF algorithm reached 93%.

The second step in this analysis, was to retrieve the feature importance from the trained model calculated as the mean decrease in impurity. The term “impurity” refers to how good the data are split in a node in the weak classifiers of the RF algorithm. Higher values indicate that the respective feature is more important, while small decrease in impurity suggests low significance for the model’s performance. To implement this step the standard python library scikit-learn v.1.13 was used.

# Supplementary Figures and Tables

Supplemental Table S1: Clinical Characteristics of EPOGH Participants by GMM Clusters

| Characteristic | Cluster 1  (n=76) | Cluster 2  (n=258) | Cluster 3  (n=84) | Cluster 4  (n=127) |
| --- | --- | --- | --- | --- |
| *Anthropometrics* |  |  |  |  |
| Females n (%) | 41 (53.95) | 140 (54.26) | 60 (71.43)& | 68 (53.54)† |
| Age, y | 30.52 ± 11.69 | 34.17 ± 13.2* | 47.72 ± 10.45*† | 47.27 ± 13.22*† |
| Body mass index, kg/m² | 22.77 ± 3.42 | 24.56 ± 4.5* | 26.76 ± 4.32*† | 27.82 ± 5.1*† |
| Systolic pressure, mm Hg | 117.13 ± 12.62 | 120.82 ± 16.0 | 131.04 ± 19.66*† | 136.22 ± 20.92*† |
| Diastolic pressure, mm Hg | 70.93 ± 7.99 | 76.26 ± 10.38* | 82.99 ± 12.01*† | 86.89 ± 12.29*†& |
| Heart rate, beats/minute | 66.79 ± 9.52 | 73.47 ± 9.72* | 69.94 ± 9.76*† | 74.41 ± 10.5*& |
| *Questionnaire data* |  |  |  |  |
| Current smoking, n (%) | 18 (23.68) | 75 (29.07) | 13 (15.48)† | 26 (20.47) |
| Drinking alcohol, n (%) | 20 (26.32) | 85 (32.95) | 23 (27.38) | 43 (33.86) |
| Hypertensive, n (%) | 6 (7.89) | 58 (22.48)* | 33 (39.29)*† | 66 (51.97)*† |
| Treated for hypertension, n (%) | 4 (5.26) | 27 (10.47) | 16 (19.05)*† | 32 (25.2)*† |
| History of cardiac disease, n (%) | 0 (0.0) | 10 (3.88) | 5 (5.95)* | 11 (8.66)* |
| History of diabetes mellitus, n (%) | 0 (0.0) | 2 (0.78) | 3 (3.57) | 13 (10.24)*† |
| *Biochemical data* |  |  |  |  |
| Serum creatinine, μmol/l | 80.35 ± 12.79 | 80.89 ± 14.64 | 81.61 ± 20.12 | 82.58 ± 14.36 |
| Total cholesterol, mmol/l | 4.57 ± 0.9 | 4.72 ± 1.05 | 5.42 ± 1.09*† | 5.39 ± 0.95*† |
| *Echocardiography* |  |  |  |  |
| LV structure |  |  |  |  |
| LV internal diameter (cm) | 4.96 ± 0.44 | 5.01 ± 0.45 | 4.95 ± 0.35 | 5.0 ± 0.49 |
| Relative wall thickness | 0.34 ± 0.04 | 0.35 ± 0.05* | 0.38 ± 0.05*† | 0.4 ± 0.07*†& |
| LV mass index (g/m) | 81.78 ± 16.69 | 85.63 ± 20.47 | 90.16 ± 18.11* | 97.19 ± 22.19*† |
| LV hypertrophy, n (%) | 3 (3.95) | 37 (14.34)* | 20 (23.81)*† | 40 (31.5)*† |
| LV systolic function |  |  |  |  |
| LV end-systolic volume index, ml/m² | 17.77 ± 5.13 | 18.93 ± 4.97 | 17.01 ± 4.69† | 18.42 ± 5.31& |
| LV end-diastolic volume index, ml/m² | 49.4 ± 10.77 | 49.95 ± 10.08 | 47.88 ± 8.95 | 48.94 ± 9.71 |
| Stroke volume index, ml/m² | 31.63 ± 6.98 | 31.02 ± 6.39 | 30.87 ± 6.12 | 30.52 ± 6.07 |
| Ejection fraction, % | 64.17 ± 5.55 | 62.24 ± 5.18* | 64.67 ± 6.11† | 62.67 ± 6.3& |
| LV longitudinal strain, % | 19.82 ± 1.71 | 18.09 ± 1.42* | 19.83 ± 1.79† | 16.57 ± 2.02*†& |
| LV diastolic function |  |  |  |  |
| E/A ratio | 1.89 ± 0.47 | 1.51 ± 0.44* | 1.09 ± 0.29*† | 0.93 ± 0.3*†& |
| e' peak, cm/s | 14.21 ± 2.67 | 12.83 ± 3.15* | 9.49 ± 2.28*† | 8.71 ± 2.91*†& |
| E/e' ratio | 5.81 ± 1.33 | 6.15 ± 1.61 | 7.79 ± 2.14*† | 7.72 ± 2.56*† |
| LV diastolic dysfunction, n (%) | 4 (5.26) | 18 (6.98) | 22 (26.19)*† | 40 (31.5)*† |
| Values are mean (±SD) or number of subjects (%). LV hypertrophy was a LV mass index of 52 g/m^2.7^ in men and 45 g/m^2.7^ in women or more. Significance for between-phenogroup differences: **P*<0.05 vs Cluster 1; †*P*<0.05 vs Cluster 2; &*P*<0.05 vs Cluster 3. A indicates late peak diastolic velocity of mitral blood flow; E early peak diastolic velocity of mitral blood flow; e’, early peak diastolic myocardial velocity; LV, left ventricular. | | | | |

Supplemental Figure S1: Risk for major adverse events by GLS quartiles. *Panel A* shows the incidence of adverse CV events. *Panel B* illustrates the Cox regression adjusted hazard ratio for CV events compared to the average population risk.


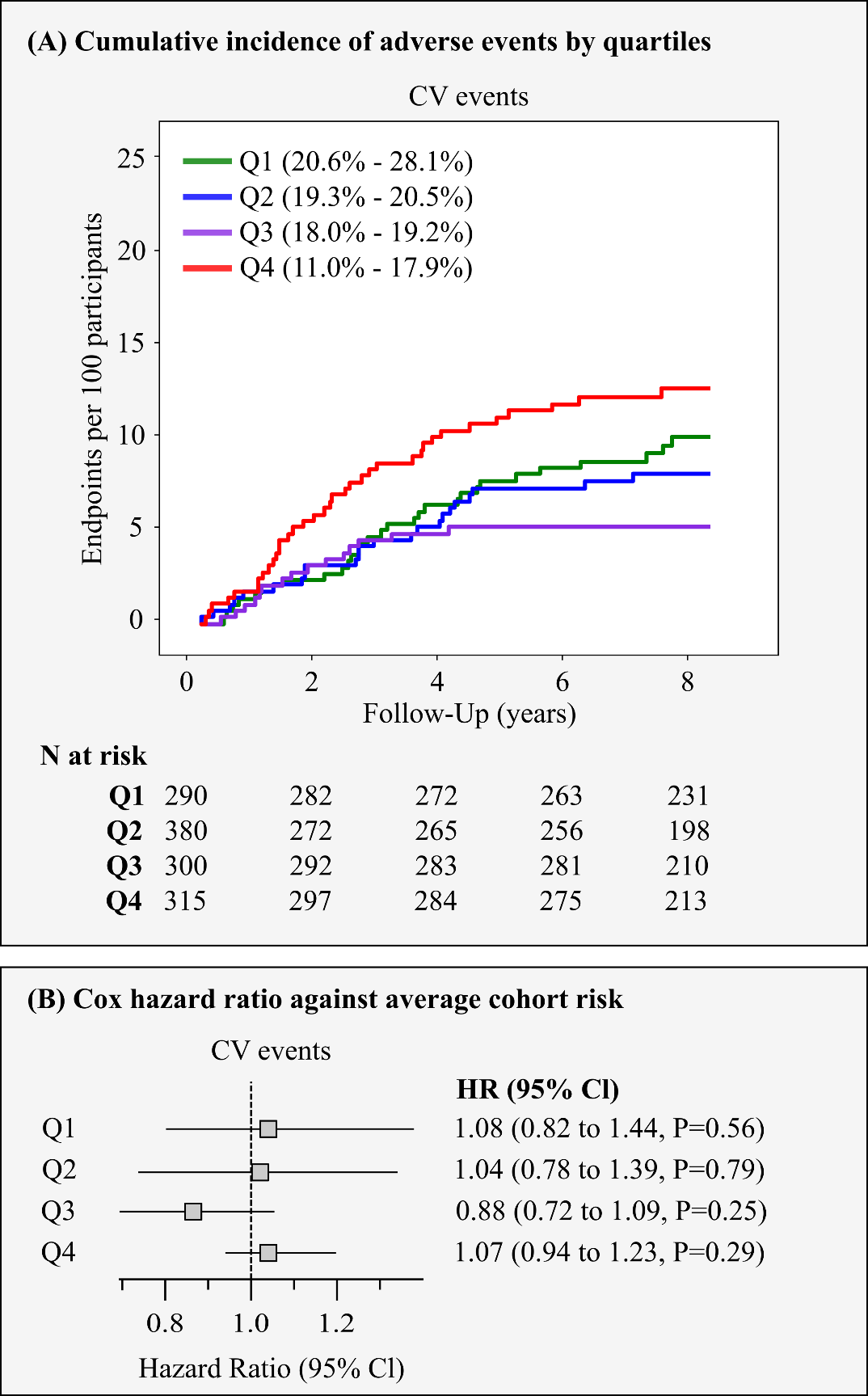

Supplement: Supplementary file 1 [file Datasheet1.docx]
